# Long-Term Follow-Up of Subjects Without Overt Heart Disease With an Early Repolarization/J Wave Electrocardiographic Pattern

**DOI:** 10.3389/fcvm.2022.831381

**Published:** 2022-02-24

**Authors:** Gaetano Antonio Lanza, Veronica Melita, Antonio De Vita, Antonio Bisignani, Roberto Mollo, Filippo Crea

**Affiliations:** Department of Cardiovascular Sciences, Fondazione Policlinico Universitario A. Gemelli IRCCS, Università Cattolica del Sacro Cuore, Rome, Italy

**Keywords:** electrocardiogram, early repolarization, J wave, clinical outcome, general population

## Abstract

**Aims:**

The “early repolarization” (ER) pattern and J wave are frequent findings on standard ECG. Controversial data have recently been reported about their prognostic implications in healthy subjects, but no longitudinal prospective study specifically designed to investigate their long-term prognostic value has hitherto been published.

**Methods and Results:**

We prospectively enrolled 4,176 consecutive subjects with no evidence of cardiovascular disease who were referred for standard ECG recording for routine check-ups or pre-operative assessments for non-cardiovascular surgery. ECGs were prospectively assessed for the presence of ER/J wave. A 10-year follow-up was available for 3,937 patients (94.3%), 660 of whom (16.8%) showed ER/J wave whereas 3,277 did not. A total of 644 deaths occurred (16.3%), 116 (2.95%) of which were attributed to cardiovascular causes. Both total and cardiovascular mortality adjusted for clinical and laboratory variables did not differ significantly between patients with vs. without ER/J wave (HR 0.94; 95% CI 0.75–1.19; *p* = 0.63 and HR 0.61; 95% CI 0.31–1.21; *p* = 0.16, respectively). No significant association with total and cardiovascular mortality was also found in pre-specified analyses for ER and J wave alone, ER/J wave detected in specific ECG regions (i.e., inferior, lateral, precordial), and type of J wave (notched or slurred).

**Conclusion:**

In this specifically designed prospective study of individuals without any evidence of cardiovascular disease, we found no significant association of ER/J wave with the risk of the total as well as cardiovascular mortality during long-term follow-up.

## Introduction

The pattern of “early repolarization” at the ECG has for a long time been considered a benign finding (1–4). A few years ago, however, some case-control studies and, subsequently, some population studies reported a significant association of early repolarization (ER) with an increased risk of sudden death (5–11), as well as cardiovascular and total mortality (9, 12–15), raising clinical and medico-legal concerns among cardiologists (16, 17). Further subsequent studies suggested that the increased risk might be related to the presence of prominent J waves rather than the typical ST-segment elevation of ER (18). Other studies, however, failed to detect any increased risk associated with ER/J wave (19–24). These controversial results have left the question of the risk associated with the ER/J wave in the population unresolved. Previous population studies, however, were not specifically designed to prospectively investigate the prognostic role of ER/J wave, but they were rather based on a retrospective assessment of ECGs of subjects included in institutional databases or enrolled in studies planned with other objectives. Furthermore, the definition of ER, J point, and J wave varied among studies.

In 2010, we started a fully prospective study, specifically designed to investigate the clinical implication of ER/J wave pattern in individuals with a demonstrated absence of any overt cardiovascular disease (25). In this study, we reported the 10-year follow-up of this cohort of subjects.

## Methods

### Subject Selection

Between January and July 2010, we enrolled, in a prospective cohort study, consecutive subjects referred to perform a routine ECG recording at our Cardiology Outpatient Clinics or our Center for pre-hospital evaluation of patients planned to undergo elective noncardiovascular surgery. The design of the study, the clinical characteristics of subjects, and the methods of ECG analysis have been described in detail elsewhere (25). Furthermore, a 6-year follow-up of a subgroup of age and sex-matched subjects with or without ER/J wave has been reported (26).

Consecutive subjects who did not have any evidence of heart disease were enrolled in the study. The absence of heart disease was initially excluded based on the absence of any symptom of possible cardiac origin (e.g., chest pain, dyspnea, syncope, etc.), the absence of any abnormal finding (e.g., arrhythmia, diastolic murmur, any systolic murmur of grade >2) on cardiac auscultation and the absence of any significant abnormality (e.g., tachy- or bradyarrhythmias, intraventricular conduction disorders, significant ST-segment and/or T wave changes, etc.) at standard ECG. In patients referring to any symptom of possible cardiac origin and/or with doubtful findings on auscultation and/or ECG, transthoracic echocardiography and maximal exercise stress test were performed, and subjects were included only if they were both normal. Subjects in whom there was evidence or even any residual doubts of heart disease (valvular, ischemic, etc.) were excluded from the study and referred for a further specific assessment.

Subjects were, in particular, excluded in case of a non-sinus heart rhythm, intraventricular conduction disturbances, pacemaker stimulation, and any other ECG finding believed to interfere with an accurate assessment of the ER/J wave pattern.

Detailed information was acquired from each subject using a standardized questionnaire. Data included age, sex, body mass index (BMI), cardiovascular risk factors, symptoms suggestive of arrhythmias, the reason for performing the ECG, drug therapy. Definitions of cardiovascular risk factors have been described in detail elsewhere (25, 26). All subjects gave written informed consent to participate in the study, which was approved by our Institutional Review Board.

### ECG Analysis

The ECGs were collected and independently analyzed by two experienced physicians to identify the presence of an ER pattern and/or J wave. Any discordance was resolved by consensus, with the supervision of a third cardiologist.

**Figure 1:**
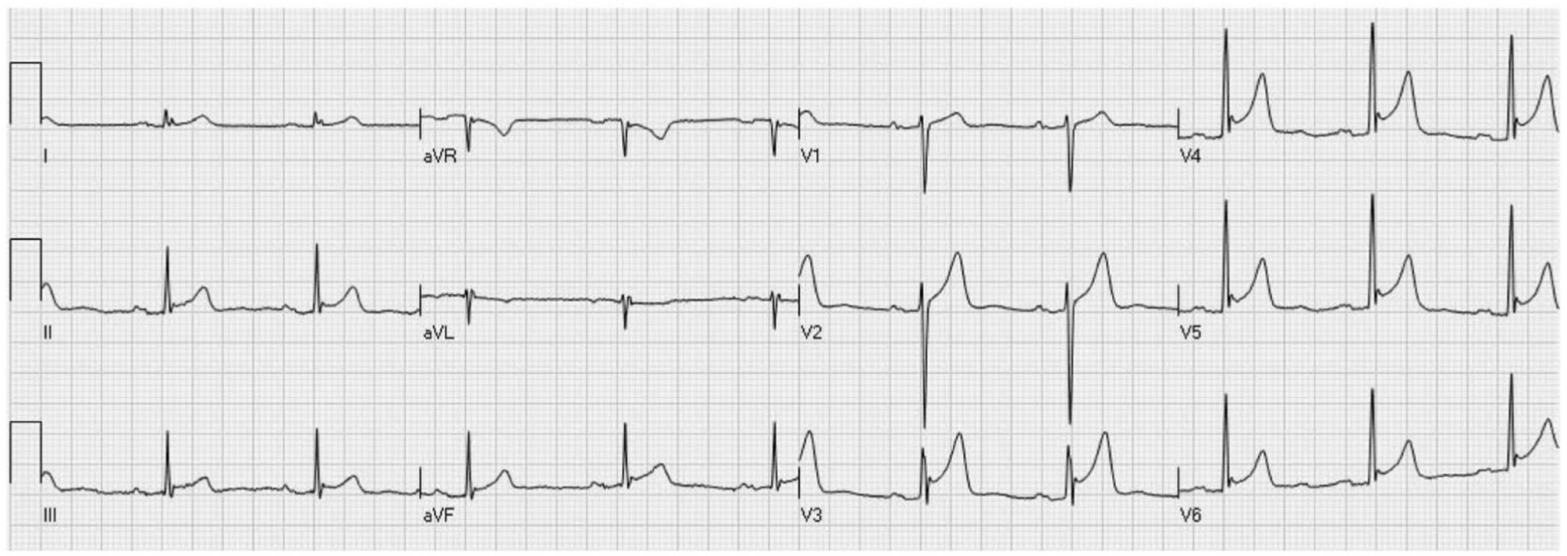
Standard ECG of a young (32 years old) healthy male subject showing a typical ST-segment elevation, with notched J wave in V4-V6 leads (with extension to V3) and, less accentuated, also in inferior leads.

The methods of ECG analysis and diagnosis of ER and J wave have previously been described in detail (25). Briefly, ER was defined as the presence of typical concave ST-segment elevation of 0.1 mV or higher in at least 2 contiguous inferior (DII-DIII-aVF), limb lateral (DI-aVL), and/or left precordial (V4-V6) leads. The J point was identified with the point where a clear linear ST-segment started and a “J wave” was diagnosed when the terminal part of the QRS, which, by definition, preceded the J point, showed a notch (notched J wave) or a widening (slurred J wave) in at least 2 contiguous leads. Specifically, a notched J wave was defined as a positive wave inscribed in the descending limb of the QRS with an amplitude >0.1 mV with respect to the isoelectric line, whereas a slurred J wave was diagnosed when the QRS showed a widening in the terminal part of the QRS which was > 20 ms in duration and >0.1 mV in amplitude with respect to the isoelectric line. As previously reported, intra- and inter-observer agreements were good for diagnosis of both ER (Cohen κ0.94 and 0.83, respectively) and J wave (Cohen κ0.97 and 0.88, respectively) (25). A typical case of the ER/J wave pattern is shown in Figure 1.

### Clinical Follow-Up

The vital status of subjects at 10 years from enrolment was ascertained by consulting the website of the Health System of Regione Lazio, Italy. For deceased subjects, the cause of death was ascertained by obtaining the ICD-9/ICD-10 codes reported in death certificates and, whenever possible, by also consulting clinical records or acquiring clinical information from subjects' relatives through telephone calls.

We planned to ascertain the relation of ER/J wave both with total death, cardiovascular death, and sudden death (i.e., unexpected death occurring within 1 h of any symptom onset or unwitnessed death). Furthermore, we assessed the relation with total and cardiovascular mortality of ER/J wave detected in different ECG locations (inferior, lateral, and precordial), the type of J wave (notched or slurred), and maximal J wave/ST-segment elevation.

### Statistical Analysis

The comparisons between groups of continuous variables were done by independent Student's *t*-test, while discrete variables were compared by the chi-square test. The association of variables with survival was evaluated by univariate Cox regression analysis. Survival curves were constructed with the Kaplan-Meier method and compared by the log-rank test. Multivariable Cox regression was applied to assess the independent association of ER/J wave with mortality endpoints when ER/J wave showed significant or borderline association (*p* < 0.1) with the outcome at univariate analysis. To this aim clinical/laboratory variables with a *p* < 0.1 at univariate analysis were included in the multivariable models. Data are reported as mean ± *SD* or numbers and percentages. A *p* < 0.05 was always required for statistical significance. Statistical analyses were performed with the SPSS 21 software (SPSS Italia, Florence, Italy).

## Results

Among 4,176 subjects included in the study, 687 (16.5%) had evidence of ER/J wave. A complete 10-year follow-up was available for 3,937 subjects (94.3%; mean age 52.1 ± 18 years; 40.7% men). Among these subjects, 660 (16.8%) showed ER/J wave whereas 3,277 did not. Among subjects with ER/J wave, 24 (3.6%) showed the classical finding of ER only (i.e., typical concave ST-segment elevation), 578 (87.6%) a J wave only, and 58 (8.8%) both.

Among the 82 subjects with ER, the pattern was found in inferior leads in 46 (56.1%), in lateral peripheral leads in 21 (25.6%), and lateral precordial leads in 67 (81.7%). The typical ER pattern was located in 1 region only in 40 subjects (48.8%), in 2 regions in 32 (39%), and all 3 regions in 10 subjects (12.2%).

Among the 636 subjects with J wave, the pattern was found in inferior leads in 428 (67.3%), in lateral peripheral leads in 203 (31.9%), and lateral precordial leads in 210 (33.2%). The J wave was located in 1 region only in 468 subjects (73.6%), in 2 regions in 132 (20.7%), and all 3 regions in 36 subjects (5.7%).

### Clinical Outcome

During the 10-year follow-up period, 644 deaths occurred (16.3%). The association of clinical variables considered in the study with total death and cardiovascular death is summarized in Tables 1, 2, respectively. Several variables were significant predictors of all-cause and/or cardiovascular death, including age, sex, heart rate, body mass index, diabetes, and hypertension.

**Table 1:** Relation of clinical and ECG variables with all-cause death.

**Variable**	**Dead (*n* = 644)**	**Alive (*n* = 3293)**	**HR (95% CI)**	***p***
Male sex	52.0%	37.9%	1.69 (1.45–1.97)	<0.001
Age (years)	68 ± 12	49 ± 17	1.08 (1.07–1.09)	<0.001
Heart rate (bpm)	71.5 ± 13	73.4 ± 14	0.99 (0.98–1.00)	<0.001
Body mass index (Kg/m^2^)	26.1 ± 5	25.6 ± 5	1.02 (1.00–1.04)	0.012
Family history of CVD	28.6%	34.5%	0.78 (0.65–0.92)	0.003
Diabetes mellitus	18.3%	10.3%	1.79 (1.47–2.19)	<0.001
Hypertension	53.5%	29.8%	2.43 (2.08–2.84)	<0.001
Smoking	18.5%	21.6%	0.83 (0.68–1.01)	0.064
Hypercholesterolemia	24.1%	23.9%	0.88 (0.82–1.18)	0.88
**Drug therapy**				
Beta-blockers	17.0%	9.5%	1.80 (1.46–2.21)	<0.001
Calcium-antagonists	9.7%	5.1%	1.82 (1.40–2.37)	<0.001
ACE inhibitors/ARBs	32.3%	18.1%	1.96 (1.66–2.31)	<0.001
Statins	10.8%	8.9%	1.19 (0.93–1.53)	0.17
Antiaggregants	18.8%	9.0%	2.06 (1.69–2.52)	<0.001
Diuretics	21.8%	8.7%	2.53 (2.10–3.06)	<0.001
Antiarrhythmics	4.2%	1.9%	1.98 (1.34–2.90)	0.001

*ACE, angiotensin converting enzyme; ARBs, angiotensin receptor blockers; CVD, cardiovascular disease; ER, early repolarization*.

**Table 2:** Relation of clinical and ECG variables with CV death.

**Variable**	**CV death (*n* = 116)**	**No CV death (*n* = 3821)**	**HR (95% CI)**	***p***
Male sex	53.4%	39.8%	1.71 (1.18–2.46)	<0.001
Age (years)	72.2 ± 11	51.3 ± 18	1.10 (1.08–1.12)	<0.001
Heart rate (bpm)	71.4 ± 12	73.4 ± 14	0.99 (0.98–1.00)	0.15
Body mass index (Kg/m^2^)	26.4 ± 4.4	25.6 ± 4.8	1.03 (1.00–1.07)	0.09
Family history of CVD	28.4%	33.7%	0.79 (0.53–1.18)	0.25
Diabetes mellitus	19.0%	11.4%	1.78 (1.12–2.83)	0.015
Hypertension	67.2%	32.6%	4.05 (2.75–5.96)	<0.001
Smoking	19.0%	21.1%	0.88 (0.55–1.30)	0.58
Hypercholesterolemia	25.9%	23.9%	1.11 (0.73–1.68)	0.63
**Drug therapy**				
Beta-blockers	24.6%	10.3%	2.71 (1.77–4.16)	<0.001
Calcium-antagonists	10.5%	5.8%	1.88 (1.03–3.42)	0.039
ACE inhibitors/ARBs	44.7%	19.6%	3.16 (2.19–4.58)	<0.001
Statins	14.9%	9.0%	1.73 (1.04–2.90)	0.036
Antiaggregants	27.2%	10.1%	3.14 (2.08–4.74)	<0.001
Diuretics	30.7%	10.3%	3.63 (2.44–5.41)	<0.001
Antiarrhythmics	10.5%	2.0%	5.07 (2.79–9.21)	<0.001

*ACE, angiotensin converting enzyme; ARBs, angiotensin receptor blockers; CVD, cardiovascular disease; ER, early repolarization*.

**Figure 2:**
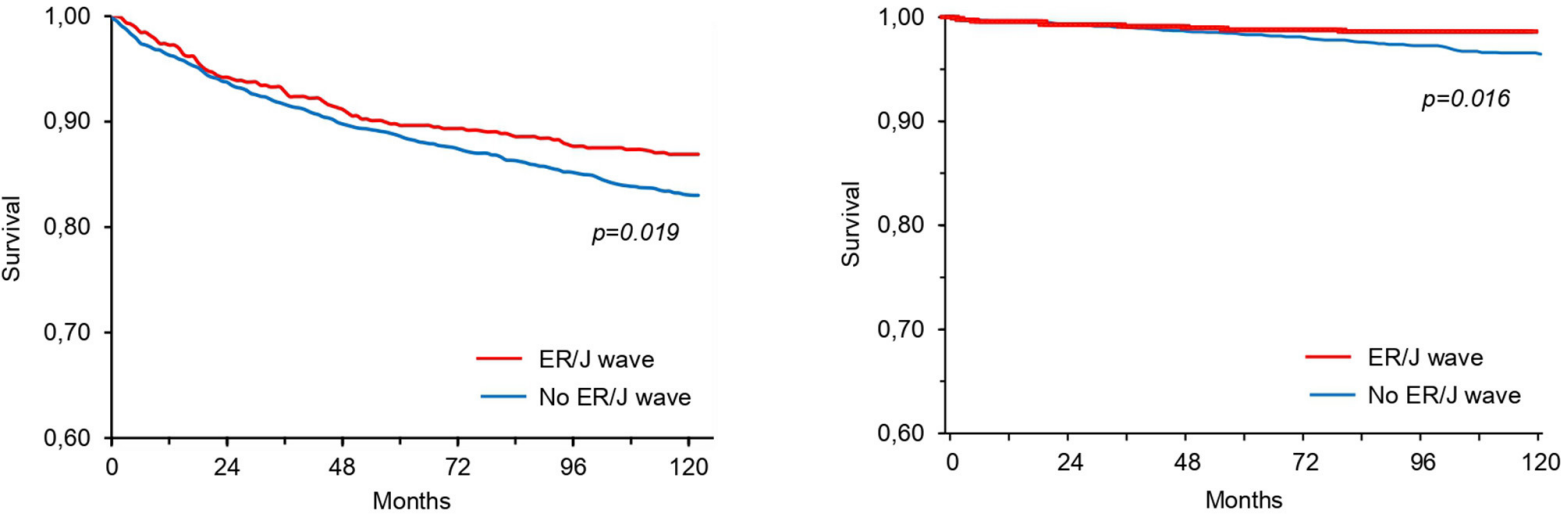
Survival curves for all-cause mortality (left) and cardiovascular mortality (right) in patients with or without ER/J wave at standard ECG (unadjusted *p*-values).

**Figure 3:**
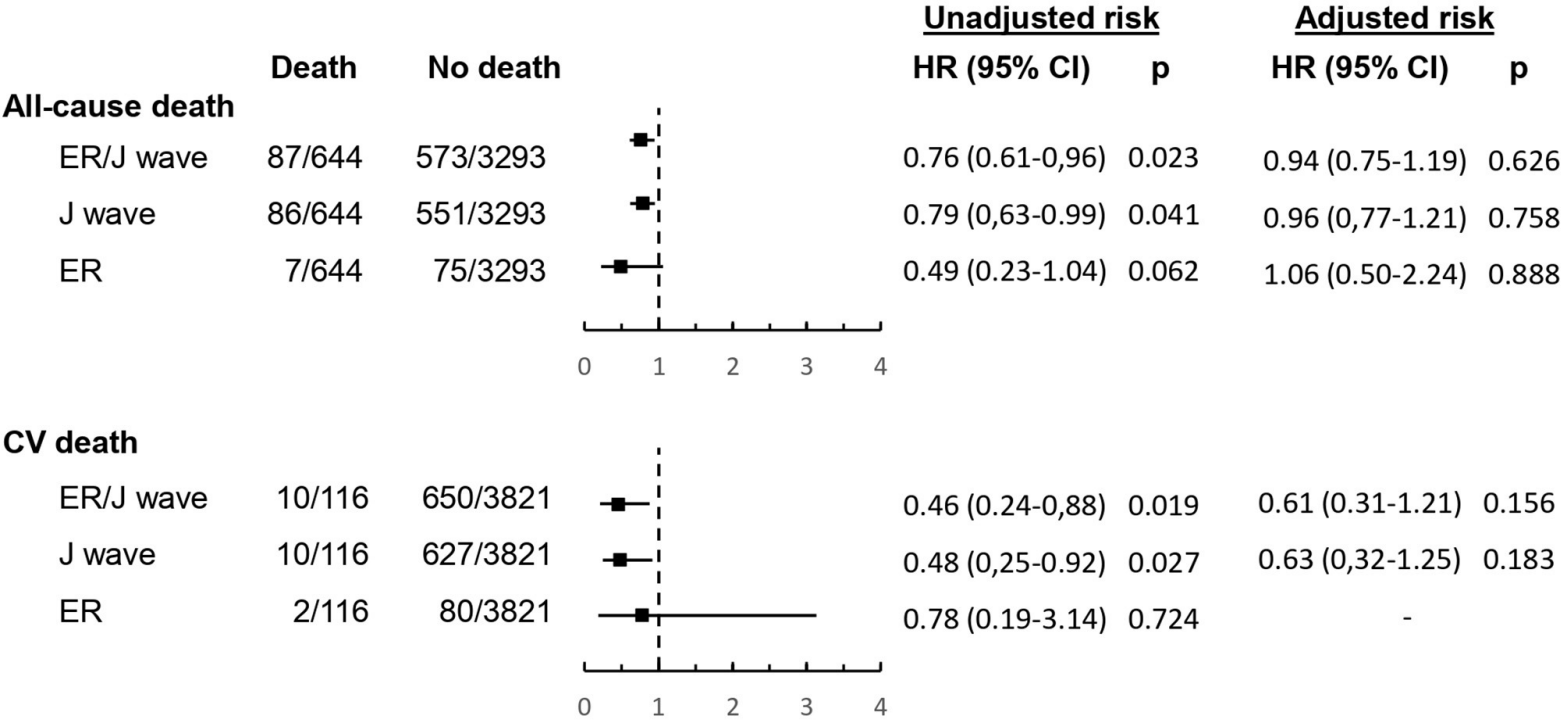
Hazard ratios (with 95 confidence intervals) for all-cause and cardiovascular mortality of ER and/or J wave. See Methods for description of statistical adjustment.

The survival curves of subjects with and without ER/J wave are shown in Figure 2, whereas the unadjusted and adjusted hazard ratios for all-cause and cardiovascular mortality are summarized in Figure 3. Deaths occurred in 87 subjects (13.2%) with and 557 (17%) without ER/J wave (*p* = 0.023). Death rate was lower in both subjects with ER (*p* = 0.062) and J wave (*p* = 0.041). However, in a multivariate analysis, the ER/J wave showed no significant association with death (Figure 3).

Death could be attributed to cardiovascular causes in 116 subjects (18% of deaths, or 2.95% of the population). One death only could be attributed to sudden death, which occurred in a subject without ER/J wave. ER/J wave (unadjusted *p* = 0.019) and J wave (unadjusted *p* = 0.027), but not ER (*p* = 0.72), were significantly associated with a lower occurrence of cardiovascular death at univariate analysis. However, in a multivariate analysis, both ER/J wave and J wave alone showed no significant association with cardiovascular death (Figure 3).

### Subgroup Analysis

**Figure 4:**
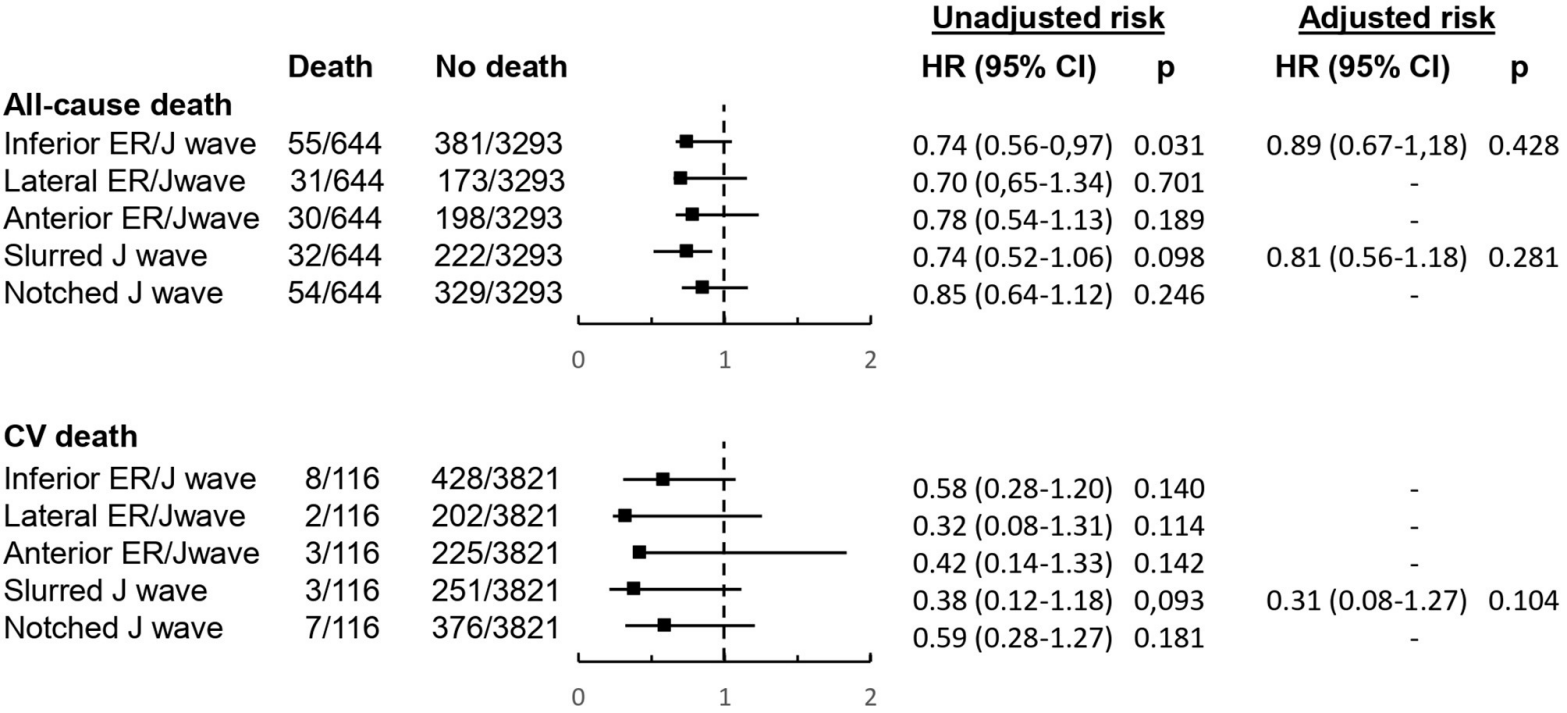
Hazard ratios (with 95 confidence intervals) for all-cause and cardiovascular mortality according to ECG location of ER/J wave and type of J wave. See Methods for description of statistical adjustment.

The assessment of ECG location (inferior, lateral, anterior) of ER/J wave and the type of J wave (slurred, notched) with both all-cause death and cardiovascular death was consistent with the main results, confirming the lack of any significant relation between ER/J wave with clinical outcome after adjustment for clinical variables (Figure 4). Also, among subjects with ER/J wave, maximal J wave and ST-segment elevation did not differ significantly between subjects with and those without fatal events (Table 3).

**Table 3:** Maximal J wave and ST-segment elevation in subjects with or without fatal events during follow-up.

	**Event**	**No event**	***p***
**Total deaths**
Maximal J wave (mm)	1.37 ± 0.52	1.37 ± 0.59	0.94
Maximal STE (mm)	1.14 ± 0.24	1.07 ± 0.24	0.45
**Cardiovascular deaths**
Maximal J wave (mm)	1.50 ± 0.71	1.37 ± 0.58	0.49
Maximal STE (mm)	1.0^*^	1.08 ± 0.25	-

* *Data referred to 2 subjects only, both with 1 mm STE*.

*STE, ST segment elevation*.

## Discussion

To the best of our knowledge, this is the first fully longitudinal long-term study assessing the prognostic role of ER/J wave in subjects without any evidence of cardiovascular disease, with baseline ECG specifically assessed for the presence of ER (i.e., a typical concave ST-segment elevation) and/or a J wave (either slurred or notched) according to clearly specified criteria (17). Our data show that in subjects without any evidence of heart disease, the presence of an ER/J wave pattern at a routine standard ECG is not associated with any significant increase in global, as well as cardiovascular, long-term mortality.

According to old studies, the detection of the ER/J wave pattern at standard ECG was traditionally considered a benign finding (1–4). However, in recent years the innocent character of this finding came into question after Haissaguerre et al. reported, in a case-control study, an increased rate of ER/J wave among patients with idiopathic ventricular fibrillation and sudden death as compared to matched healthy control subjects (5). The association of ER/J wave with arrhythmic death in otherwise healthy subjects was subsequently supported by other case-control studies (6–8) as well as population studies (9–11). Moreover, some studies suggested that ER/J wave is also associated with cardiac and/or cardiovascular deaths (9, 12–14), or even total mortality (9, 12–15).

Other studies, however, reported variable results as far as the prognostic implications of an ER/J wave pattern. Some studies, indeed, found that the increased risk was related to the presence of a prominent J wave, but not of the typical ST-segment elevation (13, 14, 18) others showed a significant association with clinical events of notched, rather than slurred, J wave, and of inferior, rather than anterolateral, J wave location (18); others found a prognostic role for ER/J wave in subgroups of individuals only (e.g., women, white, etc…) (10, 27); finally, other studies failed to find any prognostic value for ER/J wave in apparently healthy subjects (19–24).

Thus, the complexity of the available data leaves doubts about the real implications of the detection of ER/J waves in asymptomatic people. Importantly, while previous studies presented some differences in the definition of ER/J wave, end-points, reference populations, and methods of analysis that might account, at least in part, for the different conclusions, a common limitation is that they were either retrospective or, although derived from prospective cohorts, ECGs were not prospectively analyzed for the presence of ER/J wave according to pre-specified criteria.

Our study was specifically designed to prospectively assess the clinical outcome of the ER/J wave. The original population included 4,176 consecutive individuals without any apparent heart disease, the ECG tracings of whom were analyzed and prospectively classified at enrolment (25).

In the present study, we failed to find any significant association of ER/J wave with global and cardiovascular mortality at 10-year follow-up. Most important, one case of sudden death only, occurring in a patient without ER/J wave, was identified in our population, thus further questioning the arrhythmogenic potential for the ER/J wave pattern, at least in asymptomatic people without any clinical evidence of cardiovascular disease. Importantly, both the classical pattern of ER (i.e., the presence of typical ST-segment elevation) and the detection of notched or slurred J wave, as well as their ECG location, also showed no significant relation with a negative outcome.

Some limitations of our study should be acknowledged. First, the number of cardiovascular deaths was relatively low, which may not allow definitive conclusions about a potential increased risk of cardiac death associated with ER/J. However, if this were the case, it would be of questionable practical application, given the very low rate of events in this unselected population of subjects. Second, the population included in our study is not an expression of the general population of healthy people, as it included a combination of subjects without heart disease undergoing ECG for various symptoms or a check-up and patients with various surgical diseases, undergoing a pre-intervention ECG. Third, in some previous study (9, 10) the prognostic implication of ER/J wave was shown at a very long-term follow-up; thus we cannot exclude that some prognostic value of ER/J wave might emerge by prolonging the follow-up of subjects; however, it should be observed that ER/J wave decreases with increasing age (4, 25) and, therefore, it is questionable to correlate deaths occurring after many decades with ER/J wave detected at the ECG many years before, as it would not be clear whether ER/J wave persisted at the time of death (12, 15, 25). Fourth, our methods to diagnose ER and J wave are different from those proposed in a recent expert consensus document (28). However, our study was planned several times before this document was published. Whether the new detailed proposed criteria to diagnose ER/J wave may lead to different results should be assessed in prospectively designed studies.

## Conclusion

Our data showed that in subjects without any evidence of heart disease, the detection of ER and/or J wave at standard ECG is not associated with any significant increase of the risk of the total as well as cardiac death at 10-year follow-up.

## Data Availability Statement

The raw data supporting the conclusions of this article will be made available by the authors, without undue reservation.

## Ethics Statement

The studies involving human participants were reviewed and approved by the Ethics Committee Catholic University of the Sacred Heart. Written informed consent to participate in this study was provided by the participants' legal guardian/next of kin.

## Author Contributions

GL designed the study, analyzed the data, and drafted the manuscript. VM, AD, AB, and RM enrolled the patients, collected data, and handled the database. FC critically reviewed the manuscript. All authors meet ICMJE recommendation criteria for authorship in this manuscript, read and agreed to the published version of the manuscript.

## Funding

This study was supported by research funds from the Catholic University of the Sacred Heart.

## Conflict of Interest

The authors declare that the research was conducted in the absence of any commercial or financial relationships that could be construed as a potential conflict of interest.

## Publisher's Note

All claims expressed in this article are solely those of the authors and do not necessarily represent those of their affiliated organizations, or those of the publisher, the editors and the reviewers. Any product that may be evaluated in this article, or claim that may be made by its manufacturer, is not guaranteed or endorsed by the publisher.
